# T-Cell Receptor Therapy in the Treatment of Ovarian Cancer: A Mini Review

**DOI:** 10.3389/fimmu.2021.672502

**Published:** 2021-04-13

**Authors:** Jessica W. Y. Wu, Sudiksha Dand, Lachlan Doig, Anthony T. Papenfuss, Clare L. Scott, Gwo Ho, Joshua D. Ooi

**Affiliations:** ^1^ School of Clinical Sciences, Monash University, Clayton, VIC, Australia; ^2^ Walter and Eliza Hall Institute of Medical Research, Parkville, VIC, Australia; ^3^ Department of Oncology, Monash Health, Clayton, VIC, Australia

**Keywords:** T-cell receptor, tumor neoantigen, cell-based therapy, ovarian cancer, immunotherapy

## Abstract

Ovarian cancer, in particularly high-grade serous ovarian cancer (HGSOC) and ovarian carcinosarcoma (OCS), are highly aggressive and deadly female cancers with limited treatment options. These tumors are generally unresponsive to immune check-point inhibitor (ICI) therapy and are referred to as immunologically “cold” tumors. Cell-based therapy, in particular, adoptive T-cell therapy, is an alternative immunotherapy option that has shown great potential, especially chimeric antigen receptor T cell (CAR-T) therapy in the treatment of hematologic malignancies. However, the efficacy of CAR-T therapy in solid tumors has been modest. This review explores the potential of another cell-based therapy, T-cell receptor therapy (TCR-T) as an alternate treatment option for immunological “cold” OC and OCS tumors.

## Introduction—Ovarian Cancer; High Grade Serous Ovarian Cancer and Ovarian Carcinosarcoma

Ovarian cancer (OC) is the 8^th^ most commonly diagnosed cancers among women globally, with epithelial ovarian carcinomas being the most common type of OC diagnosed (1, 2). Unfortunately most OC, more than 75% of cases, are diagnosed at late stages (stage III to IV) due to their indolent presentation and the lack of effective screening tools.

**Figure 1:**
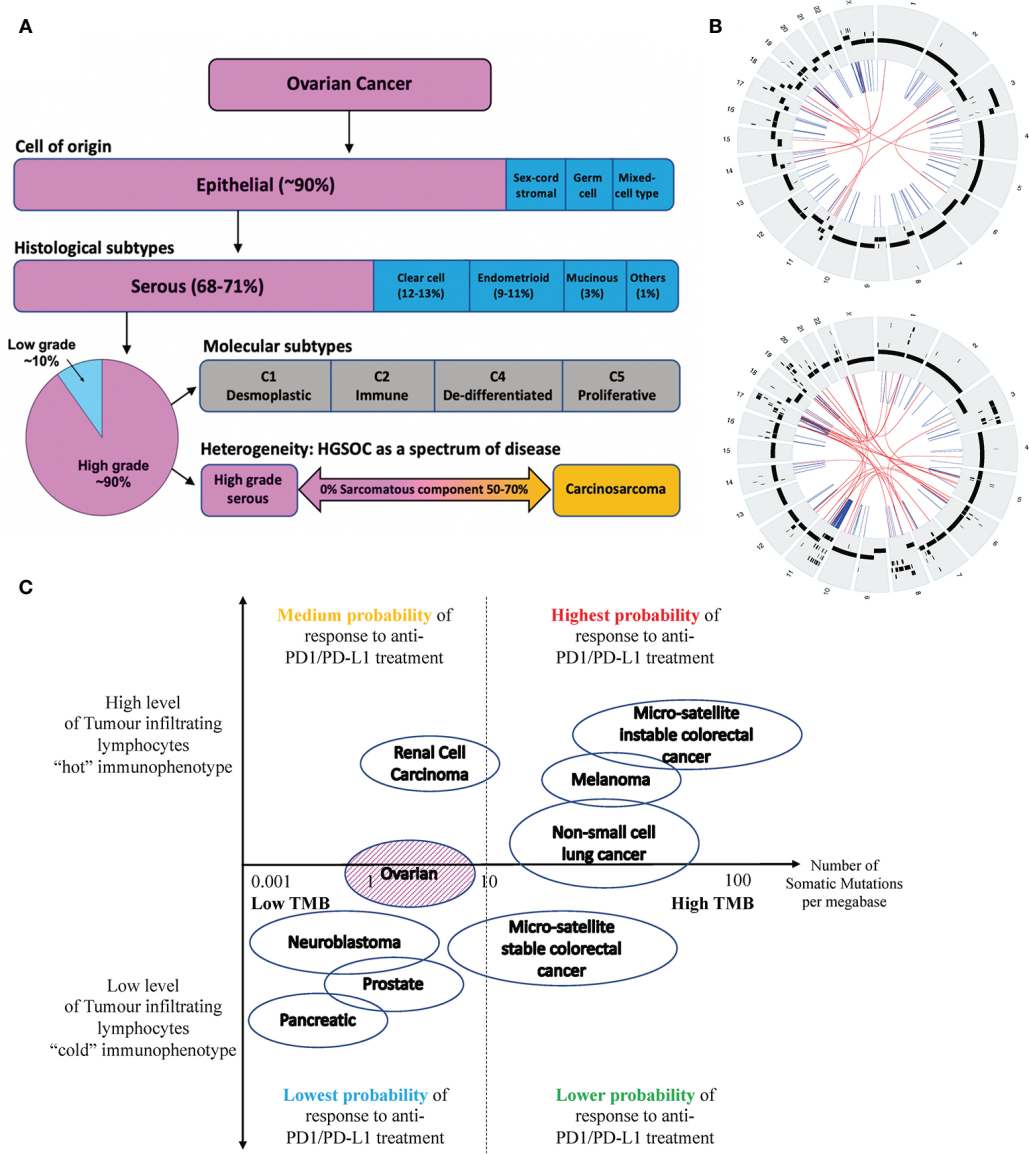
**(A)** Ovarian cancer can be classified into four subtypes based on their cell of origin and histological characteristics. Epithelial OC is the most common subtype of OC, which can be further divided into 5 histological groups. HGSOC is the most common subtype of EOC accounting for about 70% of cases and is associated with the poorest prognosis. HGSOC can be further molecularly sub-classified based on its gene expression profile, into 4 groups (C1, C2, C4 and C5). Ovarian or fallopian tube derived carcinosarcoma (O/FTCS) is a rare subtype of OC and may belong within the spectrum of HGSOC tumor. **(B)** CIRCOS plots of a patient with HGSOC (top) and a patient with OCS (bottom) with low tumor mutation burden – demonstrating a high degree of chromosomal instability **(C)** Schematic diagram of tumors with high and low tumor mutation burdens versus inflamed and non-inflamed immunophenotypes based on their T-cell and IFN signature. Although some ovarian cancers may have low TMB, these tumors may still respond to ICI if they are associated with a “hot” immunophenotype. Completely “cold” tumors are those associated with low TMB and cold immunophenotype features, such as pancreatic, prostate cancer and some OCS.

The majority of epithelial ovarian carcinomas are high-grade serous ovarian carcinomas (HGSOC), which are of aggressive nature and associated with poor 5-year overall survival of less than 35 percent (2) (see **Figure 1A**). HGSOC is a heterogenous group of cancers that can be molecularly subtyped based on their gene expression profile (3). Ovarian carcinosarcomas (OCS) are a rare subtype of OC with a worse prognosis (4). It is argued that OCS may be a subtype of HGSOC given that they share common genomic features and cell of origin (5).

Improvements in survival outcomes for OC patients worldwide over the past two decades have been minimal, with the exception of the recent introduction of Poly (ADP-ribose) polymerases inhibitors (PARPi) (6). In recent years, *in vivo* pre-clinical testing of novel therapeutics for OC have been aided by an improved understanding of tumor biology – built upon decades of technological advancements in genetic engineering and refinements in mouse models, including the patient-derived xenograft (PDX) model.

Currently, cell-based immuno-oncology for solid tumors such as in OC is an emerging field, which holds a wealth of unrealized potentials. This review will provide an overview of cell-based immunotherapies for OC, in particular T-cell receptor cell-based immunotherapy approach targeting tumor neoantigens (TNA) to treat immunologically “cold” HGSOC and OCS.

## High-Grade Serous Ovarian Cancer and Ovarian Carcinosarcoma Are Often Regarded as Immunologically “Cold” Tumors

### Immunotherapies (IO), in Particular, Immune Checkpoint Inhibitors (ICI), Are Often Ineffective When Delivered as a Single Agent in OC, Particularly in HGSOC

Immunotherapies are increasingly being explored as a potential treatment option for OCs, given the longevity of responses observed in responsive tumors (7–9). These responsive tumors are often referred to as immunologically “hot” tumors. ICIs are a traditional class of immunotherapies used to target tumors that disrupt the immune checkpoint pathways for immune evasion. ICIs such as anti-PD-1/PDL-1 monoclonal antibodies (e.g., nivolumab, pembrolizumab) targeting the PD-1/PD-L1 axis or anti-CTLA-4 antibodies (e.g., ipilimumab) have shown a great degree of clinical efficacy as single agent or in-combination in non-small cell lung cancer and melanoma respectively with the list of cancer subtypes that are being or under investigation is rapidly expanding (currently including head and neck carcinoma, renal cell carcinoma, uterine carcinoma, colorectal and upper gastro-intestinal carcinoma, bladder carcinoma, hepatocellular carcinoma and small cell lung cancer) (10, 11). These therapies are associated with acceptable toxicities reported in multiple Phase II/III clinical trials (12).

However, ICI efficacy is limited in HGSOCs. These tumors are often not mutation-driven and tend to have low rates of tumor mutation burden (TMB) (13–16).. The use of single agent ICIs has resulted in modest response rates in most clinical trials involving HGSOC, however, those who responded, achieved significant clinical benefit. Hence, these findings serve to inform and outline the importance of selecting the right tumor groups within HGSOC for ICI, in particular identifying biomarkers to predict ICI responses. Another approach to improve the response rate is to use ICI with a combinatory synergistic partner such as in combination with a PARPi or a mitogen-activated protein kinase kinase inhibitor (MEKi) (17, 18).

Due to OCS rarity, the efficacy of IO, in particularly ICI, is relatively unknown. Given that OCS share similar genomic landscape with HGSOC, it is likely that OCS is also relatively immunologically “cold” (19).

### HGSOC and OCS Are Often Regarded as “Class C” Tumors Where Tumorigenesis Is Driven by Chromosomal Instability Rather Than Mutation

Almost all HGSOCs demonstrate the loss of maintenance of their genome integrity leading to extreme genomic instability (20). This is demonstrated by the remarkable degree of genomic disarray seen in all these tumors characterized by high level of genomic structural variability, associated with frequent somatic copy number alterations (SCNA), in both gains and losses (21–23). Therefore, these tumors are classified as “Class C” malignancy, together with a large fraction of lung (LUSC) and head and neck (HNSC) squamous cell carcinoma and endometrioid tumor of the serous subtype (UCEC-serous) (24). The “Class C” tumors in general have high levels of chromosomal instability as well as enrichment of TP53 mutations, which in turn contribute to the tumor genomic instability. The opposite is the “Class M” tumor, which is predominantly driven by mutation.

The genomic instability is highly relevant for HGSOC tumorigenesis because chromosomal structural change is one of the important mechanisms for tumor suppressor gene (TSG) inactivation either through heterozygous or homozygous loss (25), or gene breakage (26). Similarly, high gene copy number gain can result in differential expression of oncogenes, such as *MYCN* and *CCNE1*, which are relevant in the initiation of tumorigenesis and as mechanisms of drug resistance (3, 26–28).

Therefore, HGSOC tumorigenesis driven by loss of TSG and/or over-expression of oncogenes, which are normal genes with specific function in normal tissue whose expressions are well regulated within non-malignant cells. Hypothetically, the loss of TSG and over-expression of oncogenes should not make these tumors immunogenic. These tumorigenic processes do not result in expression of abnormal mutant proteins or peptides that would be recognizable by the body’s immune system.

OCS tumorigenesis is thought to be based on the “conversion hypothesis” of sarcomatous transformation of a differentiated cancer cell-type (carcinoma) into undifferentiated (sarcomatous) malignancy (29, 30). It is generally accepted that the transformation is not driven by somatic mutation, but *via* reprogramming of gene expression and associated with high level of genomic instabilities (see **Figure 1B**). In keeping with HGSOC, OCS should also not be immunogenic.

### Tumor Mutational Burden Is Often Low in HGSOC (and/or OCS) and May Be One of the Reason for the Immunological “Cold” Phenotype

Tumor mutational burden (TMB) is defined as the total number of somatic mutations per coding area of a tumor genome (31). TMB has been regarded as an emerging potential clinical biomarker associated with response to immune checkpoint inhibitor (ICI) therapy (32). A higher TMB is commonly observed in cancers associated with mutagens, such as ultraviolet light exposure in melanoma and smoking in non–small-cell lung cancer (NSCLC). The prevalence of high TMB was seen in approximately 52% of patients with melanoma and 38% to 42% of patients with NSCLC. High TMB or TMB-H is often defined as detection of more than 10 mutations per megabase of DNA, denoted as mut/Mb. In general sense, tumors with high TMB are likely to express more neoantigens that may be one of the factors driving anti-tumor immunity thus making these tumors “hot” and more responsive to anti-PD-1/PD-L1 therapies (33). It is important to take into consideration that ICI therapies mode of action is not to initiate T-cells activities but to re-invigorate already tumor-reactive T-cells (33).

In contrast, due to the drivers underlying HGSOC and OCS tumorigenesis, HGSOC and OCS are often associated with lower TMB or TMB-L. Tumors with TMB-L have been linked to poorer responses to ICI. In contrast, TMB-H tumors are associated with better responses to ICI as they potentially have higher levels of neoantigens that can be recognized by the immune system (32, 33). However, not all TMB-L tumors are immunologically “cold” as the presence of tumor infiltration lymphocytes (TILs) or inflamed T-cells may improve tumor response to ICI – see **Figure 1C**.

There are a number of other factors that have been proposed to contribute to the “cold” tumor immune-phenotype in HGSOC. These include the expression of endothelin B receptor (34) and Fas ligand (35) within the tumor endothelium, elevated levels of vascular endothelial growth factors (VEGF) which are commonly seen in HGSOC (36), and the presence of epigenetic silencing of Th1-type chemokines (37). The overexpression of endothelin B receptor, Fas ligand and VEGF altered the tumor micro-environment, in particularly affecting the tumor endothelial barrier to hinder T-cell homing and infiltration into the tumor. Similarly, the repression of Th1-type chemokines expression, such as CXCL9 and CYCL10, leads to impaired T-cell trafficking into the tumor. The reduction or absence of TILS compounds the lacks of ICI activity. Cold OC tumors are also shown to be enriched for a class of genomic alterations known as foldback inversions, resulting in high level of chromosomal translocation (38).

However, not all OC or HGSOC are cold immunologically (see **Figure 1C**). Those HGSOC that are immunologically “hot” are associated with mutations in *BRCA1* and, in some studies, *BRCA2* (39, 40). Studies have shown that within immunological cold tumors, there are tumor sites which showed enhanced signs of immune editing, including neoantigen depletion and allele-specific loss of MHC class I, suggesting that these tumors were immunologically “hot” to begin with (38).

## Adoptive T-Cell Therapies (ATC); A Potential Approach To Treat Immunologically “Cold” Ovarian Cancers

### Cell-Based Therapy May Be an Alternative Immunotherapy Option for Immunologically “Cold” OCs

Cell-based therapy strategy includes the adoptive transfer of autologous antigen-specific T-cells, which have undergone *ex vivo* modification and expansion, with the aim of achieving a targeted immune response (41). This can be achieved with the utilization of *ex vivo* expanded tumor infiltrating lymphocytes (TIL) with high tumor-specific reactivity, or genetically modified peripheral blood mononuclear cells (PBMC) (41).

Earlier attempts have been relatively unsuccessful due to various challenges posed by these approaches in solid tumors, mainly the small number of invasive TILs within immunologically “cold” tumors and the lack of anti-tumor ability of the body’s immune system (41). However, recent advances have shown that the low survival and migration of T cells can be overcome, and immune evasion, at least in hematological malignancies, can be circumvented by T-cell genetic engineering (42).

With respect to generating ATC with improved OC antigen specificity, peripheral blood lymphocytes can be genetically modified to express: (i) a T cell receptor (TCR) with specificity for a tumor-restricted peptide presented by a given HLA molecule (43, 44), or (ii) a “chimeric antigen receptor” (CAR) comprising an antigen-binding domain (typically derived from an antibody) linked *via* a transmembrane domain to one or more intracellular signaling domains derived from the T cell receptor complex and associated co-stimulatory molecules (45).

### Chimeric Antigen Receptor T Cell Therapy Is a Well-Developed Approach to Treat Hematologic Malignancies

Chimeric antigen receptors (CARs) redirect T cells to recognize cell surface antigens in an HLA-independent manner. By doing so, it has the potential of becoming an “off-the-shelf” universal approach to treat wide range of tumors expressing the appropriate cell surface antigens. To achieve this, CAR-T cells utilize antibody fragments that bind to specific antigens on the surface of cancer cells.

CAR-T cell therapy has been well studied and is approved for the treatment of various hematological malignancies (38). These include the treatment of relapsed or refractory B-cell precursor acute lymphocytic leukemia (ALL) in patients ≤25 years old (46) approved by the Food and Drug Agency (FDA) in August 2017 and the treatment of adults with relapsed or refractory large B-cell lymphoma, including diffuse large B-cell lymphoma (DLBCL), after two or more lines of systemic therapy (47).

Despite CAR-T therapy successes in hematologic malignancies, the efficacy in solid tumors is less dramatic and is confounded by the associated risk of cytokine release storm (CRS) and other significant immunologic toxicities (48, 49). Unlike for hematological malignancies, it is harder to justify the use of CAR-T in solid tumors due to its much lower response rates and associated high cost. Despite this, there are multiple on-going or completed clinical trials of CAR-T therapies in OC targeting MUC1, MUC16, mesothelin, or folate receptor α (50).

### T Cell Receptor Therapy Is an Alternate Cell-Based Therapy With Great Potential for Treatment of Solid Tumors

T cell receptors (TCRs) use T-cell antigen receptors, which consist of alpha and beta chains, to recognize polypeptide fragments presented by major histocompatibility complexes (MHC) molecules (51). The generation of TCR-T involves a transfer of TCR genes from an activated T-cell to a naïve T-cell. In doing so, the TCR binding to tumor antigens can be genetically modified to enhance specificity and affinity to desired cancer antigens (51). Unlike CAR-T therapy, whose target antigens are only cell surface proteins, TCR-T cell therapy can recognize intracellular antigen fragments as well as surface proteins as long as these are presented by MHC molecules. However, this also means that TCR-T cell therapy is MHC restricted and depends on the presentation by MHC molecules to recognize targets and activate T cell function. There lies both the advantages and disadvantages of TCR-T over CAR-T therapies (49). Furthermore, the somatic loss of HLA-1 in immunologically “cold” tumor will also have a negative impact on the efficacy of TCR-T (52).

CD8+ cytotoxic T cells play crucial role in the killing of cancerous or virally infected cells. The CD8+ T cells that are used in TCR-T therapy retain all their natural auxiliary molecules of the TCR signal transduction pathway. Therefore, TCR-T cells can be fully activated even when a very small amount of antigen is present, resulting in effective antigen-specific killing (51).

Furthermore, there are many downstream co-stimulatory factors involved in TCR signaling, including the upregulation of anti-apoptotic factors such as B cell lymphoma 2 (BCL-2), BCL extra-large (BCL-xL) and BCL2-related protein A1 (BCL2A1). These anti-apoptotic factors promote T cell activation and more importantly their survival during this process (53). Although, there are also confounding co-inhibitory factors within the T-cell system to extinguish T cell signaling in order to keep their cytotoxic activities in check, such as cytotoxic T-lymphocyte antigen 4 (CTLA-4) and programmed cell death protein 1 (PD-1), these co-inhibitory factors can be circumvented with anti-PDL-1 and anti-CTLA-4 compounds that are widely available in the clinical settings (49).

Lastly, the bystander effect is largely unappreciated in cell based therapy in solid tumor. It has been observed that significant numbers of T cells are activated in a T cell receptor-independent and cytokine-dependent manner, a phenomenon referred to as “bystander activation” during T-cell cytotoxic process. The mechanisms of the bystander effect are unclear, but the innate inflammatory cytokines, such as IL-18 and IL-15, are thought to play crucial roles for inducing bystander activation during infection (54). Bystander activation leads to host injury mediated by exerting a higher level cytotoxicity that is further facilitated by natural killer cell-activating receptors, such as NKG2D, and cytolytic molecules, such as granzyme B. Therefore, hypothetically a small number of activated CD8+ T cells infiltrating into a “cold” tumor may exert both T cell receptor dependent and independent cytotoxic effects on the cancer cells. There is evidence that the bystander effect has been demonstrated to be less profound in relation to CAR-T in solid tumor seen in syngeneic mouse cancer models that is not augmented by co-administration of anti-PD-1 or anti-CTLA-4 agents (55).

## T-Cell Receptor Transduced T Cell Therapy in Ovarian Cancer: Current Therapeutic Landscape

T-cell receptor transduced-T cell (TCR-T) therapy in OC is currently in early phase clinical trials. There are currently well-documented OC markers and targets for TCR therapy, in particular cancer-testis antigens (CTA). These CTAs include melanoma-associated antigen 4 (MAGE-A4) and New York esophageal-1 (NY-ESO-1) (56–60).

CTAs are a group of proteins important for early organogenesis and as developmental proteins. Their expressions are highly regulated and are often switched off in adulthood, with the exception of the male germ cell population and in certain subset of cancers (61). Due to this, in particularly their tumor-restricted expression, and their abilities to induce strong *in vivo* immunogenicity, CTAs are now regarded as ideal targets for tumor specific IO approaches.

The Melanoma-associated antigen (MAGE)-A gene family is a group of CTA genes that encode for MAGE-A1, MAGE-A2, MAGE-A3, MAGE-A4, MAGE-A6, MAGE-A10, and MAGEA12 (51, 62). These were the earlier targets for TCR-T and are expressed at a frequency of about 1/10 000. Unfortunately, there were cross-reactivities seen in TCR-T specific to MAGE-A peptides to related peptides in the brain and the heart. These cross-reactivities have resulted in severe immune related toxicities reported in TCR-T early phase clinical trials which included severe organ damages (such as the brain and heart) and even death (63).

NY-ESO-1 or New York esophageal squamous cell carcinoma 1 is another well-known CTAs where its expression is usually restricted to testicular germ cells and placenta trophoblasts. There was no or low expression seen in normal adult somatic cell, with re-expression observed in numerous cancer types including ovarian cancers (64)

As these CTAs are not entirely private but can still be found in normal somatic tissues, targeting these may result in minor to detrimental off-target toxicities. Furthermore, these listed CT antigens are not mutagenic proteins but re-expression of normal developmental proteins in cancer cells. In addition, these markers are not always exclusively upregulated in every OC, resulting in variable response rates. **Table 1** listed all the Phase I and II clinical trials that are recruiting women with OC to assess the safety profiles of TCR-T.

**Table 1:** Summary of ongoing clinical trials for evaluating TCR therapy safety profiles in cancers including OC.

Study ID and description	Study phase	Intervention	Target
NCT03017131 (“Genetically Modified T Cells and Decitabine in Treating Patients With Recurrent or Refractory Ovarian, Primary Peritoneal, or Fallopian Tube Cancer”)	I	TCR therapy	NY-ESO-1
NCT03691376 (“Genetically Engineered Cells (NY-ESO-1 TCR Engineered T Cells and HSCs) After Melphalan Conditioning Regimen in Treating Patients With Recurrent or Refractory Ovarian, Fallopian Tube, or Primary Peritoneal Cancer”)	I	TCR therapy	NY-ESO-1
NCT01567891 (“CT Antigen TCR-redirected T Cells for Ovarian Cancer”)	I/IIa	TCR therapy	NY-ESO-1
NCT02650986 (“Gene-Modified T Cells with or Without Decitabine in Treating Patients with Advanced Malignancies Expressing NY-ESO-1”)	I/IIa	TCR therapy	NY-ESO-1
NCT02096614 (“Investigator Initiated Phase 1 Study of TBI-1201)	I	TCR therapy	MAGE-A4
NCT03412877 (“Administration of Autologous T-cells Genetically Engineered to Express T-cell Receptors Reactive Against Mutated Neoantigens in People with Metastatic Cancer”)	II	TCR therapy	Unspecified

TCR, T-cell receptor; NY-ESO-1, New York esophageal-1; MAGE-A4, melanoma-associated antigen 4.

## Potential Future Role of TCR-T Therapy in Immunologically Cold Rare Tumor and The Concept of Personalized Therapies Targeting Tumor Neoantigens

TCR-T approach may be an attractive cell based therapy option for immunological “cold” tumor given the wider repertoire of targetable tumor neoantigens (compared to CAR-T cell based therapy). Despite having low TMB, each HGSOC and OCS will have tumor neoantigens (TNA), which can be identified by next generation sequencing of the tumors. There are a wider array of TNA targetable by TCR-T, as they are not restricted to the peptides presented on the surface of cancer cells, but also internal TNA as long as these are associated with high affinity to the patient’s human leukocyte antigen (HLA) class I complexes. Thus, these will be presented to CD8+ T-cytotoxic cells.

The selection of the target TNA should not be affected by the heterogeneity or clonality of the tumor, although the selection of a truncal TNA is preferable, as tumor infiltrating activated T cells will exert a degree of bystander cytotoxic effect - a phenomenon that is well documented in CD8+ T cell activity in viral infections. The more crucial step to the process of developing a TNA-specific T cell therapy is the identification of a TNA-specific TCR. Once the TNA-specific TCR is identified, the TCR can be transduced into patient derived autologous CD8+ T cells.

TNA selection, TCR identification and *ex vivo* genetic engineering are the key components for a successful personalized TCR-T in immunologically cold tumors, such as OC and OCS. At present, there are now technologies that enable the modification of the patient’s own CD4+ and CD8+ cells’ TCRs to express specific peptide enhanced affinity receptors (SPEARs) to increase the binding affinity of natural TCRs, thus overcoming their low affinities as a result of negative thymic selection during maturation of T cells in the thymus (51). Lastly, the reducing cost of Next Generation Sequencing will allow for faster, cheaper TNA discovery and may pave the way for personalized TCR-T.

## Author Contributions

GH, JO, and JW contributed to conception and design of the manuscript. GH and JW wrote the first draft of the manuscript. GH, CS, AP, SD, LD, and JO wrote sections of the manuscript. JW, SD, and LD contributed to the figure. All authors contributed to the article and approved the submitted version.

## Funding

GH is funded by a School of Clinical Sciences, Monash University Clinician-Scientist Early Career Fellowship. JO is an Al and Val Rosenstrauss Fellow funded by the Rebecca Cooper Medical Foundation. Clare Scott is funded by the Stafford Fox Medical Research Foundation (CLS); Cancer Council Victoria (Sir Edward Dunlop Fellowship in Cancer Research to CLS); the Victorian Cancer Agency (Clinical Fellowships to CLS CRF10–20, CRF16014.

## Conflict of Interest

The authors declare that the research was conducted in the absence of any commercial or financial relationships that could be construed as a potential conflict of interest.
